# Prediction of psychosis: model development and internal validation of a personalized risk calculator

**DOI:** 10.1017/S0033291720004675

**Published:** 2022-10

**Authors:** Tae Young Lee, Wu Jeong Hwang, Nahrie S. Kim, Inkyung Park, Silvia Kyungjin Lho, Sun-Young Moon, Sanghoon Oh, Junhee Lee, Minah Kim, Choong-Wan Woo, Jun Soo Kwon

**Affiliations:** 1 Department of Psychiatry, Seoul National University College of Medicine, Seoul, Republic of Korea; 2 Department of Psychiatry, Pusan National University Yangsan Hospital, Yangsan, Republic of Korea; 3 Department of Brain and Cognitive Neuroscience, Seoul National University College of Natural Sciences, Seoul, Republic of Korea; 4 Center for Neuroscience Imaging Research, Institute for Basic Science, Suwon, Republic of Korea; 5 Department of Biomedical Engineering, Sungkyunkwan University, Suwon, Republic of Korea

**Keywords:** Clinical high-risk (CHR), LASSO, personalized medicine, prediction, psychosis, transition

## Abstract

**Background:**

Over the past two decades, early detection and early intervention in psychosis have become essential goals of psychiatry. However, clinical impressions are insufficient for predicting psychosis outcomes in clinical high-risk (CHR) individuals; a more rigorous and objective model is needed. This study aims to develop and internally validate a model for predicting the transition to psychosis within 10 years.

**Methods:**

Two hundred and eight help-seeking individuals who fulfilled the CHR criteria were enrolled from the prospective, naturalistic cohort program for CHR at the Seoul Youth Clinic (SYC). The least absolute shrinkage and selection operator (LASSO)-penalized Cox regression was used to develop a predictive model for a psychotic transition. We performed *k*-means clustering and survival analysis to stratify the risk of psychosis.

**Results:**

The predictive model, which includes clinical and cognitive variables, identified the following six baseline variables as important predictors: 1-year percentage decrease in the Global Assessment of Functioning score, IQ, California Verbal Learning Test score, Strange Stories test score, and scores in two domains of the Social Functioning Scale. The predictive model showed a cross-validated Harrell's *C*-index of 0.78 and identified three subclusters with significantly different risk levels.

**Conclusions:**

Overall, our predictive model showed a predictive ability and could facilitate a personalized therapeutic approach to different risks in high-risk individuals.

## Introduction

Over the past two decades, early detection and early intervention in psychosis have become essential goals of psychiatry (Birchwood, Todd, & Jackson, 1998; McGlashan & Johannessen, 1996; McGorry, Killackey, & Yung, 2008). Only a small proportion of cases undergo the transition to psychosis, and the process often takes place over very long periods of time; these circumstances are among the main reasons why the focus of high-risk studies has shifted from the traditional or genetic high-risk model to the clinical high-risk (CHR) model. The CHR concept has emerged to describe cases that are likely to progress to psychosis soon (Yung et al., 1996). A number of prospective cohort programs have been introduced for help-seeking people who have not yet developed psychosis (Cannon et al., 2008; Nelson et al., 2013; Riecher-Rossler et al., 2007; Ruhrmann et al., 2010); additionally, various terms, such as at-risk mental state and ultra-high-risk, in addition to CHR for psychosis, and basic symptoms have been established to describe this population (Fusar-Poli et al., 2013; Schultze-Lutter, Schimmelmann, Ruhrmann, & Michel, 2013; Yung, Fusar-Poli, & Nelson, 2012). However, the incidence rate of psychosis in a CHR individual decreases over time (Yung et al., 2007). Only one-fourth of CHR patients develop psychosis within 3 years (Fusar-Poli et al., 2012, 2015), and there are even studies that report an incidence rate of <15% (Addington et al., 2011b; Katsura et al., 2014; Koike et al., 2013; Pruessner et al., 2017), although this risk state is not pluripotential but is specific to psychosis (Webb et al., 2015; Woods et al., 2018). Moreover, one-third of CHR patients remit from the risk state (Simon et al., 2013), and they showed no cognitive impairment or have good functional outcomes (Glenthoj, Kristensen, Wenneberg, Hjorthoj, & Nordentoft, 2020; Lee et al., 2014b). On the other hand, other non-converters also have attenuated psychotic symptoms or low levels of functioning even if they do not develop psychosis (Addington et al., 2011a; Lee et al., 2014a; Lin et al., 2015). Thus, the CHR state is a heterogeneous clinical syndrome, only a small percentage is converted to psychosis and also develops other conditions than psychosis, and for this reason, the dilution of the pretest risk of psychosis due to intensive, predominantly general-population-oriented outreach campaigns and a high rate of self-referrals has been discussed (Fusar-Poli, Schultze-Lutter, & Addington, 2016b; Fusar-Poli et al., 2016c; Mitter, Nah, Bong, Lee, & Chong, 2014). Therefore, the ability to identify true-positive patients who will later develop psychosis can immensely broaden our understanding of the pathophysiology of the long-term course of schizophrenia, and it will deepen the phenomenological, biological, and causal understanding of schizophrenia (Bentall, Jackson, & Pilgrim, 1988; Fusar-Poli & Schultze-Lutter, 2016; Guloksuz & van Os, 2018).

Predictive medicine is a discipline that entails predicting the probability of a disease's incidence or prognosticating its course, thus reducing the uncertainty in clinical decision making (Fusar-Poli, Hijazi, Stahl, & Steyerberg, 2018; Steyerberg, 2008; Wasson, Sox, Neff, & Goldman, 1985). In contrast to classical statistics, predictive modeling has high clinical utility in that it not only indicates the average characteristics of the patient's group but also provides rich information about the onset or trajectory of illness at the individual level (Braitman & Davidoff, 1996; Hahn, Nierenberg, & Whitfield-Gabrieli, 2017; Lee, Bang, & Kim, 2016). Furthermore, with the very high levels of clinical heterogeneity arising from phenotype-based diagnosis, the application of a clinical prediction model to real-world situations would facilitate better decision making in psychiatry. However, clinical impressions are insufficient for predicting psychosis outcome in CHR cases (Nelson & Yung, 2010); more rigorous and quantitative prediction models are needed. Thus, risk stratification has been applied in CHR studies (Fusar-Poli et al., 2016a, 2016b; Nieman et al., 2014; Ruhrmann et al., 2010; Schmidt et al., 2017), this is similar to the model applied in heart failure and stroke (Goldman et al., 1996; Janes, Pepe, & Gu, 2008; Lip, Nieuwlaat, Pisters, Lane, & Crijns, 2010). Moreover, a number of prediction studies have been conducted (Addington et al., 2019; Oliver et al., 2020; Studerus, Ramyead, & Riecher-Rossler, 2017), and the least absolute shrinkage and selection operator (LASSO) model, which incorporates machine learning techniques, has been introduced into such studies (Addington et al., 2017; Ciarleglio et al., 2019) to overcome the problem of overfitting (Koutsouleris, Upthegrove, & Wood, 2019; Nelson, Yung, & McGorry, 2019; Tibshirani, 1996; Tibshirani, 1997). More recently, several web-based personalized risk calculators have been developed (e.g. riskcalc.org/napls, psychosis-risk.net, link.konsta.com.pl/psychosis) (Cannon et al., 2016; Fusar-Poli et al., 2017; Kotlicka-Antczak et al., 2019), and their scope of use is being expanded through internal and external validation (Carrion et al., 2016; Fusar-Poli et al., 2019b; Osborne & Mittal, 2019; Zhang et al., 2019). These individual-level risk measures can be applied in real-world clinical practice to quantify the risks that patients may face and to develop appropriate treatment strategies. However, several additional considerations must be addressed to increase the predictive accuracy of personalized risk prediction models. First, to increase the accuracy of prediction, a modeling process based on longer-term follow-up is required to reduce false negatives that have not yet been developed due to insufficient observation period. Studies with a follow-up period of <3 years tend to have an incidence of less than one-quarter (Addington et al., 2011b; Katsura et al., 2014; Koike et al., 2013; Morrison et al., 2007; Pruessner et al., 2017; van der Gaag et al., 2012), whereas those of more than 3 years tend to have an incidence of more than one-quarter (Nelson et al., 2013; Schultze-Lutter, Klosterkotter, & Ruhrmann, 2014; Spitz et al., 2017; Ziermans et al., 2014), with some exceptions (An et al., 2010; Armando et al., 2015), although meta-analytic results indicated that the incidence of psychosis in high-risk groups peaks within the first 2 years after entry (Kempton, Bonoldi, Valmaggia, McGuire, & Fusar-Poli, 2015). A sufficient period of observation will reveal the results of false negatives who have not yet developed and enable more accurate modeling. Second, CHR samples have different risks for psychosis, depending on their referral sources. For example, samples such as those recruited from the community via the Prodromal Questionnaire–brief form and other self-report measures, as well as patients who visit a primary clinic with non-prodromal symptoms and are suspected of having attenuated psychotic symptoms, would have different pretest risk levels than help-seeking individuals visiting a secondary or specialized clinic for CHR patients. This suggests that the diversification of referral sources and the rise of intensive outreach activity are major contributors to the increased variance of pretest risk enrichment in CHR samples and that the risk of developing psychosis is becoming more heterogeneous. Given this combination of factors, samples recruited by a fixed strategy at a single institution may allow greatly improved models to be developed if external validation can be conducted.

The primary goal of this study was to develop a model for predicting the 10-year risk of psychotic transition in patients who visited the Seoul Youth Clinic (SYC) at Seoul National University Hospital. A second goal was to perform internal validation of the resulting prediction model and stratify the CHR sample by risk level.

## Methods

### Participants

Two hundred and twenty-two help-seeking CHR individuals (aged 15–35 at baseline) were enrolled in the prospective, naturalistic cohort program for prodromal psychosis at the SYC between November 2004 and November 2019 (Kim et al., 2012; Kwon, Byun, Lee, & An, 2012; Lee et al., 2014a). All participants were recruited mainly from a psychiatric outpatient clinic in the Seoul National University Hospital and made initial contact with the SYC via the website (http://www.youthclinic.org). All subjects were diagnosed with the Korean version of the Structural Interview for Prodromal Syndrome (SIPS) and belonged to at least one of CHR groups: attenuated positive symptoms (APS), brief intermittent psychotic symptoms (BIPS), and genetic risk with deterioration (GRD) (Jung et al., 2010). The exclusion criteria for all subjects were as follows: any current or lifetime Axis I psychotic disorder or substance dependence other than cigarette smoking, past or present neurological disease or traumatic brain injury with loss of consciousness, a significant medical condition that could manifest as a psychiatric condition, past or current use of antipsychotic medications to manage attenuated psychotic symptoms, and/or an intelligence quotient (IQ) below 70. Clinical and cognitive function assessment was conducted by psychiatrists and clinical psychologists at 6-month intervals for the first 2 years and at 1-year intervals afterward for up to 10 years (online Supplementary Table S1). All subjects received case management and supportive psychotherapy monthly from psychiatrists. If the development of psychosis was suspected during these treatment sessions, the subjects also received an additional assessment session within a week, even during the regular evaluation period. The transition to psychosis was determined if the subject met the Presence Of Psychotic Syndrome (POPS) criteria of SIPS (Jung et al., 2010). Of the overall sample, 14 cases were excluded due to withdrawal; the final sample consisted of 208 CHR individuals. Written informed consent forms were obtained from all subjects, and if they were under the age of 18 years, guardian consent was also obtained. The present study was approved by the Institutional Review Board of the Seoul National University Hospital.

### Clinical and cognitive assessments

Depressive and anxious symptoms were evaluated using the Hamilton Depression Rating Scale (HAM-D) (Yi et al., 2005) and the Hamilton Anxiety Rating Scale (HAM-A) (Hamilton, 1959), respectively. The Global Assessment of Functioning (GAF) was administered to assess the current levels of overall symptoms and functioning (Yi, Chung, Lee, & Lee, 2003), and the percentage drop over the past year was calculated. Social functioning was also assessed using the Korean version of the Social Functioning Scale (SFS), which consists of seven domains of social behavior: social engagement/withdrawal, interpersonal behavior, independence – performance, independence – competence, recreation, prosocial activities, and employment/occupation (Kim & Lee, 2009).

Each subject's IQ was estimated using the Korean version of the Wechsler Adult Intelligence Scale-III (WAIS) (Yeom, Park, Oh, & Lee, 1992). We implemented four subsets of WAIS consisting of the Vocabulary, Arithmetic, Block Design, and Picture Arrangement, and assessment was performed by a trained researcher who majored in psychology with a master's degree or higher, or by a skilled clinical psychologist. The following neuropsychological tests were administered to assess cognitive function in the CHR population: Digit span, a subset of the WAIS, was used to measure attention/working memory. Processing speed was assessed with the Trail Making Test Part A (TMT-A) (Reitan, 1958). Measures of divided attention were assessed with the Trail Making Test Part B (TMT-B) (Reitan, 1958) and set-shifting from perseverative errors in the Wisconsin Card Sorting Test (WCST) (Chelune & Baer, 1986). Verbal fluency was evaluated with the Korean version of the verbal fluency task for semantic fluency (Kim et al., 2013) and the Controlled Oral Word Association Test (COWAT) for phonemic fluency (Kang, Chin, Na, Lee, & Park, 2000). Verbal memory was assessed with the Korean version of the California Verbal Learning Test (K-CVLT), for which we examined the sum of the immediate and delayed scores (Kim & Kang, 1999). The sum of the immediate and delayed scores on the Rey–Osterrieth Complex Figure Test (ROCF) was used to evaluate visual memory (Shin, Park, Park, Seol, & Kwon, 2006).

### Statistical analysis

All analyses were conducted in Stata version 16 (Stata Corp.) and R version 3.6.0. (Comprehensive R Archive Network). Comparisons of baseline demographic and clinical characteristics were performed with χ^2^ tests and independent *t* tests. The cumulative incidence rates of transition to psychosis during the follow-up period were estimated with the Kaplan–Meier analysis. In developing the predictive model for psychosis, the LASSO-penalized Cox regression was used to developing the predictive model with subjects who had different follow-up periods (Simon, Friedman, Hastie, & Tibshirani, 2011). Predictors included a total of 56 candidate variables, including base demographic, clinical, and cognitive variables. Missing data were handled using the multiple imputation method with *k* = 10. We selected the LASSO model that resulted in minimal prediction error using 10-fold cross-validation. We then conducted a bootstrap test with 1000 iterations to estimate the 95% confidence interval of the predictive performance. For internal validation, a predictive individual prognostic index (PI) was generated, and *k*-means clustering analysis was performed to stratify the risk of transition to psychosis using the LASSO model with the elbow method to determine the optimal *k*. Kaplan–Meier analysis was performed to estimate the incidence of psychosis in each cluster. Then, the log-rank test was used to determine the different survival functions.

## Results

The total SYC sample consisted of 208 CHR participants. The follow-up time ranged from a minimum of 30 days to a maximum of 12 years (online Supplementary Table S1). The mean follow-up duration was 3.5 years (s.d. 2.6 years). Thirty-eight participants developed a psychotic disorder during the follow-up period. Table 1 shows a Kaplan–Meier estimate of the survival function for the time to transition to psychotic disorders. The total cumulative incidence rate of transition was 32.6% (95% CI 21.8–46.9). Table 2 presents the baseline demographic and clinical characteristics of the participants. There was no significant difference between the groups in demographic status. Converters had higher positive symptom scale scores on the Scale of Prodromal Symptoms (SOPS), a percentage of changes in GAF scores in 1 year, and lower estimated current IQ, CVLT, and Strange Stories task scores. The social engagement/withdrawal and prosocial domains of the SFS showed trend-level differences between groups.

**Table 1.** Kaplan–Meier estimates of transition rates over 10 years

Time from baseline (year)	Estimated transition rate % (95% CI)	Cumulative no. of transitions
1	7.3 (4.4–12.0)	18
2	12.1 (8.1–17.9)	22
3	17.6 (12.6–24.3)	30
4	18.3 (13.2–25.2)	31
5	21.1 (15.4–28.5)	34
6	24.7 (17.7–33.7)	36
7	24.7 (17.7–33.7)	36
> 8	32.6 (21.8–46.9)	38

**Table 2.** Demographic and clinical characteristics of the participants

	Converter (*N* = 38)		Non-converter (*N* = 170)		Statistical analysis	
	*N*	%	*N*	%	χ^2^	*p*
Male/female	26/12		116/54		0.001	0.982
High/low parental socioeconomic status^a^	15/23		66/104		0.006	0.941
Subgroup					1.473	0.479
BIPS	1	2.6	11	10		
APS	35	92.1	144	81.2		
GRDS	2	5.3	15	8.8		
Family history of psychosis	9	23.7	26	15.3	0.593	0.441
Urbanicity					3.815	0.148
City	29	76.3	129	75.9		
Small town	9	23.7	28	16.5		
Rural area	0	0	13	7.6		
Tobacco use	4	10.5	20	11.8	0.047	0.829
Presence of religion	20	52.6	67	39.4	2.231	0.135
Completion of military service	8	21.1	36	21.2	0.001	0.987
Unemployment	10	26.3	33	19.4	0.903	0.342
Right handedness	34	89.5	166	90	0.01	0.922
	Mean	s.d.	Mean	s.d.	*T*	*p*
Age (years)	21.3	4.6	21.2	4.1	0.139	0.889
Education (years)	12.5	1.8	12.7	2	0.569	0.57
SOPS						
Positive symptoms scale	11.4	3.5	9.9	3.9	2.151	**0.033**
Negative symptoms scale	12.2	5.6	13.1	6.3	0.818	0.415
Disorganized symptoms scale	4.8	3	4.1	2.8	1.384	0.168
General symptoms scale	6.8	4	7.6	4	1.207	0.229
HAM-D	12.3	6.1	12.6	7.4	0.489	0.625
HAM-A	10.4	5.6	10.9	7.2	0.353	0.724
GAF	52.4	7.7	53.9	9.5	0.921	0.358
% of changes in GAF scores in 1 year	24.5	11.8	17.1	13.3	3.126	**0.002**
SFS						
Social engagement/withdrawal	93.1	11.6	96.2	9.4	1.76	0.079
Interpersonal behavior	97.1	13.4	100.9	15.5	1.403	0.162
Independence – performance	93.6	12.6	94.6	13.3	0.429	0.668
Independence – competence	95.5	12.1	97.6	16.1	0.783	0.435
Recreation	92.9	12.1	96.1	13.2	1.337	0.183
Prosocial activities	98.5	2.2	102.8	13.7	1.751	0.081
Employment/occupation	116	9.9	113.9	9.7	1.205	0.229
Estimated current IQ	101.1	11.5	106.6	14.3	2.141	**0.034**
Digit span	11.5	2	12.1	2.6	1.392	0.166
TMT-B	74.7	28.1	67.4	24.4	1.612	0.108
CVLT	20.9	5.3	23.7	5.7	2.739	**0.007**
RCFT	24.8	5.9	23.9	5.8	0.834	0.405
TMT-A	28.1	10	26	10.2	1.159	0.248
Category fluency task	29.9	10.6	31	12.3	0.53	0.597
COWAT	30.8	13.5	33.8	14.2	1.208	0.228
WCST perseverative error	10.6	5.7	9.8	5.8	0.772	0.441
Block design	12.6	2.4	12.7	2.9	0.208	0.836
Strange Stories task	19.9	3.5	21.3	3	2.501	**0.013**

BIPS, Brief Intermittent Psychotic Syndrome; APS, Attenuated Psychosis Symptoms Syndrome; GRD, Genetic Risk and Deterioration Syndrome; SOPS, Scale of Prodromal Symptoms; HAM-D, Hamilton Depression Rating Scale; HAM-A, Hamilton Anxiety Rating Scale; GAF, Global Assessment of Functioning; SFS, Social Functioning Scale; CVLT, California Verbal Learning Test; ROCF, Rey–Osterrieth Complex Figure Test; TMT, Trail Making Test; COWAT, Controlled Oral Word Association Test; WCST, Wisconsin Card Sorting Test.

a Scores of 1–3 indicate high status and scores of 4–5 indicate low status.

**Fig. 1.**
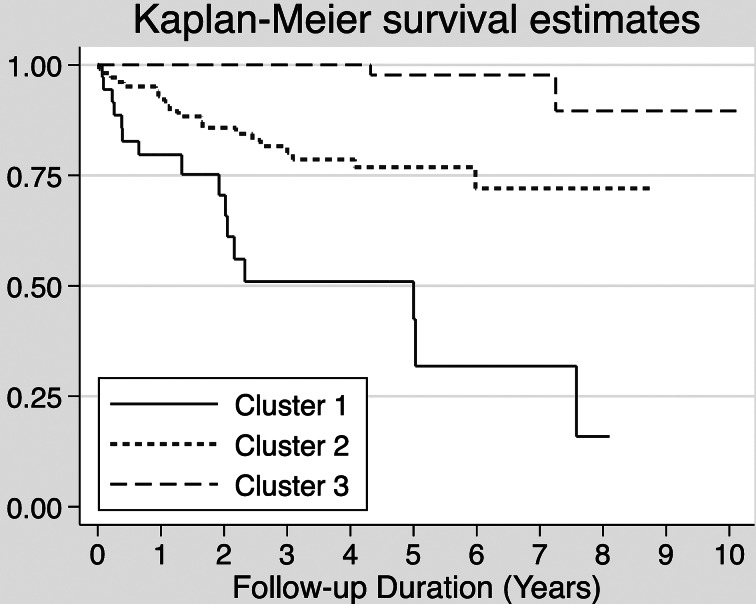
Kaplan–Meier survival estimates for the three clusters. Cluster 1 is a high-risk subgroup with 84.1% incidence (*n* = 36), cluster 2 is a medium-risk subgroup with 27.9% incidence (*n* = 109), and cluster 3 is a low-risk subgroup with 10.4% incidence within 10-year follow-up.

In the SYC sample, 7.4% of the data overall were missing. The ROCF scores had the highest amount of missing data, with 24.5% of values missing. Category verbal fluency and COWAT scores had 21.2% of values missing. The LASSO Cox prediction model identified six baseline variables, including a percentage of changes in GAF scores in 1 year, IQ, CVLT, Strange Stories task, and two domains of the SFS (Table 3). The prediction model had a cross-validated Harrell's *C*-index of 0.78 (95% CI 0.777–0.783). The optimal number of clusters for the stratification of risk using the elbow method was 3. Figure 1 shows a Kaplan–Meier estimate of the survival function for the time to transition to psychotic disorders for each cluster. The cut-off values for each cluster were −1.86 for cluster 1 and −2.33 for cluster 3. The 10-year survival rate for each cluster was 15.9% for cluster 1 (*n* = 36), and 72.1% for cluster 2 (*n* = 109), 89.6% for cluster 3 (*n* = 99), and the clusters showed a significant difference in survival rate (cluster 1 *v*. 2: χ^2^ = 37.06, *p* < 0.001, cluster 2 *v*. 3: χ^2^ = 9.1, *p* = 0.003). Patients in cluster 1 showed an approximately 44% risk of developing psychosis at 3 years, whereas none of the patients in cluster 3 developed psychosis before 3 years (Table 4; online Supplementary Table S2).

**Table 3.** The baseline variables identified by the LASSO Cox model that significantly predicted transition to psychosis

Predictor	Unstandardized coefficient	Standardized coefficient
CVLT^a^	−0.067	−0.067
Strange Stories task	−0.066	−0.068
% of changes in GAF^b^ scores in 1 year	0.042	0.041
IQ	−0.022	−0.021
Social engagement/withdrawal in SFS^c^	−0.028	−0.027
Prosocial activities in SFS^c^	−0.019	−0.019

a California Verbal Learning Test.

b Global Assessment of Functioning.

c Social Functioning Scale.

**Table 4.** Kaplan–Meier estimates of transition rates in three clusters

Time from baseline (year)	Estimated transition rate (%)		
	Cluster 1	Cluster 2	Cluster 3
1	16.6	9.4	0
2	21.8	18	0
3	43.5	23.8	0
4	43.5	25.4	0
5	52.9	27.2	2.1
6	64.7	27.2	7.3
7	64.7	27.2	7.3
>8	82.3	27.2	15

## Discussion

This study aimed to develop and internally validate a model for predicting the incidence of psychosis in CHR individuals to provide useful assistance in clinical practice. We developed a model that includes social functioning, social cognition, functional decline, verbal memory, and IQ; this model demonstrated fair predictive ability. Using this model, we divided the high-risk groups into three clusters, all of which showed significant differences in the incidence of psychosis. To the best of our knowledge, this is the first study using modern machine learning techniques to model a wide range of variables covering demographic, clinical, and cognitive functions in long-term cohorts spanning more than 10 years.

In this study, approximately 32% of subjects developed to psychosis during up to 10 years of follow-up. This transition rate is somewhat higher incidence compared to the results of a meta-analysis with an incidence rate of 20% (Fusar-Poli et al., 2016a). Although half of the total incidence occurs within the first 8 months of the 2-year follow-up period (Fusar-Poli et al., 2016a), in a long-term follow-up result of Nelson et al, 17% of cases are converted during the follow-up period after 3 years, and 5% are converted after 5 years (Nelson et al., 2013). This is in line with the result of our cohort, which showed a conversion rate of 21% after 3 years and 10% after 5 years. In our predictive model with a long-term follow-up duration, the estimated predictive ability had a *C*-index of 0.78, which is comparable to the results of other previous studies with similar designs (Addington et al., 2017; Ciarleglio et al., 2019), but never meaningfully higher. Addington et al. tracked 172 subjects for 2 years and reported that 29 subjects were converted, while Ciarleglio et al. followed 199 subjects for 2 years and 64 transitions (Addington et al., 2017; Ciarleglio et al., 2019). Given this, it can be assumed that long-term follow-up periods need not be mandatory to increase the predictive power of the model. However, since we have not yet performed external validation of this model, it will be necessary to examine whether the predictive accuracy of our model can be replicated in an independent sample with long-term follow-up duration. In regard to predictive models using the LASSO method, Addington et al. externally validated their model (Addington et al., 2017; Cornblatt et al., 2015). Of course, predictive models developed using classical statistical techniques have been externally validated and have undergone refinements several times (Fusar-Poli et al., 2019a, 2019b; Osborne & Mittal, 2019; Zhang et al., 2018), but it will be necessary to continuously refine the model through the optimization of coefficients and variable selection using the LASSO technique in the same sample. More recently, an advanced dynamic prognostic model that combines demographic and clinical variables to forecast the development of psychosis was introduced (Studerus, Beck, Fusar-Poli, & Riecher-Rossler, 2020). This model can keep up with changes in symptoms over time, allowing the model to be updated at various time points as the patient is followed. In addition to the prediction models for CHR, an alternative perspective model has also been developed and validated for those who have not yet been diagnosed with CHR (Fusar-Poli et al., 2017, 2019b). This model can be applied to a large number of populations in the community. Therefore, in the future, it will be necessary to develop appropriate models that reflect the characteristics of the sample at each stage, such as patients who show attenuated symptoms but have not yet been diagnosed with CHR, those who have just been diagnosed with CHR, and those who are being followed up.

Moreover, based on the results of the clustering analysis, our predictive model classified the CHR into three subgroups characterized by different levels of risks. Risk stratification has already been implemented several times (Addington et al., 2017; Michel, Ruhrmann, Schimmelmann, Klosterkotter, & Schultze-Lutter, 2014; Ruhrmann et al., 2010). Ruhrmann et al. first classified the samples into four PIs (Ruhrmann et al., 2010). At 18 months, the incidence of class I was 3.5%, and that of class IV was 85.1%. They also developed a new predictive model that was divided into four classes using diagnostic criteria and cognitive function (Michel et al., 2014). In this study, the cumulative hazard rate of class I was 0, and that of class IV was 1.29. However, these studies were not cross-validated; thus, it is difficult to ignore the risk of overfitting problems. Recently, Addington et al. introduced a cross-validated predictive model (Addington et al., 2017). They divided CHR into three risk groups, but there was no data on the incidence of each group. In contrast, in our model, we found that the three clusters that were stratified using the LASSO model had significantly different degrees of risk. Interestingly, in cluster 1, approximately half of the cases transitioned to psychosis within 3 years of follow-up, whereas in cluster 3, no transition occurred within that time period. Moreover, unlike simply distinguishing between the converters and the non-converters, there were a distinct demographic, symptom, and cognitive differences in each subgroup (online Supplementary Table S3). Our result will help facilitate a personalized therapeutic approach to different degrees of risks among high-risk individuals and will enrich future recruitment efforts, such as targeting only CHR patients with moderate to high risk.

Consistent with previous studies, our model included general functioning, social cognition, social functioning, and verbal memory as significant predictors (Cannon et al., 2016; Cornblatt et al., 2015; Malda et al., 2019; Zhang et al., 2019). However, demographic and prodromal symptom variables were not significant in our analyses and were not included in our model. A recent meta-analysis revealed that the demographic variables of subjects are significant predictors of transition to psychosis. In this study, each of the predictors was stratified into groups ranging from convincing evidence (class I) to weak evidence (class IV) (Oliver et al., 2020). However, out of 26 putative risk/protective factors, there were no class I predictors, while only global functioning and attenuated positive psychotic symptoms are in line with previous findings that the dynamic functional change, rather than the static functional status at a single timepoint, better predicts prognosis (Tarbox et al., 2013; Velthorst et al., 2013; Zhang et al., 2019). Similarly, we observed no significance for the SOPS score. Unlike our model, some other predictive models include both cognitive function and clinical symptoms (Addington et al., 2017; Cornblatt et al., 2015). However, these studies used the rescaled SOPS scores for their models and not the raw scores, which may have resulted in the discrepancy of the results. Further investigation into whether the cognitive function is more influential and essential than clinical symptom scores for predicting psychosis is needed.

The present study has several limitations. First, we did not utilize the variables that were acquired during the patient follow-up, including pharmacotherapy, cognitive-behavioral therapy, and compliance, in our model. Our primary goal was to predict the risk of psychosis using only baseline information. This is reasonable, as at the time of the patients' first evaluation, no interventions had been administered. Although we used 10-year follow-up data, we assumed that this long duration might compensate for the delay in the transition to psychosis related to the use of medication, a dynamic model that reflects both the baseline and longitudinal change may be a better way to solve this problem in the future (Studerus et al., 2020; Yuen et al., 2018). Second, external validation of the model was not performed in our current study. For external validation, the variables used in the model should be identical. However, the actual tasks used to assess specific cognitive domains may differ from study to study. To overcome this problem, a model needs to be developed that utilizes only the variables that overlap across studies. Third, this study was conducted as a data-driven study. Given the number of variables being injected, the sample size may not be large enough. This is a frequently mentioned issue in clinical prediction. Even with the application of data science technique to the current study, it is still probable that the model developed could be biased against the sample's characteristics. It is still necessary to be conducted with the selection of variables based on hypotheses and the development of a model. Last, it has not been explored what life events have affected the transition to psychosis. Further research on this will be needed, especially since the appraisal of life experiences is an important axis of the bio-physico-social model of schizophrenia (Garety et al., 2015).

In summary, we developed a predictive model with 10-year follow-up data of CHR patients. One-third of the CHR patients developed psychosis over a sufficient follow-up period. Our model showed that verbal ability, social cognition, social functioning, functional decline, and general intelligence are important predictors of the transition to psychosis. CHR sample was divided into three clusters according to the degree of risk. We believe that our model could facilitate a personalized therapeutic approach to different risks in high-risk individuals.

## Supplementary material

For supplementary material accompanying this paper visit https://doi.org/10.1017/S0033291720004675. click here to view supplementary material
