# Supplementary material for: Prediction of psychosis: model development and internal validation of a personalized risk calculator
Source: Psychol Med. 2020 Dec 14;52(13):2632–40. doi: 10.1017/S0033291720004675 (PMC9647536; doi:10.1017/S0033291720004675)
Supplement: Supplementary file 1 [file S0033291720004675sup001.docx]

Supplementary Table 1. Flow chart showing the assessment process for the subjects

|  | Baseline | 6 month | 1 year | 18 month | 2 year | 3 year | Thereon after |
| --- | --- | --- | --- | --- | --- | --- | --- |
| Demographics | O |  |  |  |  |  |  |
| Clinical assessments | O | O | O | O | O | O | O |
| Cognitive assessments | O |  | O |  | O | O | O |
| Self reports | O | O | O | O | O | O | O |

Supplementary Table 2. Life table for CHR sample during a 10-years follow-up.

| Interval | | Beg. Total | Deaths | Lost | Survival | Std. Error | 95% Conf. Int. | |
| --- | --- | --- | --- | --- | --- | --- | --- | --- |
| 0 | 1 | 208 | 14 | 31 | 0.9273 | 0.0187 | 0.8803 | 0.9563 |
| 1 | 2 | 163 | 8 | 21 | 0.8786 | 0.0244 | 0.8212 | 0.9185 |
| 2 | 3 | 134 | 7 | 10 | 0.831 | 0.029 | 0.765 | 0.8798 |
| 3 | 4 | 117 | 2 | 12 | 0.816 | 0.0303 | 0.7476 | 0.8675 |
| 4 | 5 | 103 | 2 | 23 | 0.7981 | 0.0322 | 0.7261 | 0.8531 |
| 5 | 6 | 78 | 3 | 40 | 0.7569 | 0.0383 | 0.6718 | 0.8228 |
| 6 | 7 | 35 | 0 | 13 | 0.7569 | 0.0383 | 0.6718 | 0.8228 |
| 7 | 8 | 22 | 2 | 4 | 0.6812 | 0.0614 | 0.5443 | 0.7848 |
| 8 | 9 | 16 | 0 | 13 | 0.6812 | 0.0614 | 0.5443 | 0.7848 |
| 9 | 10 | 3 | 0 | 2 | 0.6812 | 0.0614 | 0.5443 | 0.7848 |
| 10 | 11 | 1 | 0 | 1 | 0.6812 | 0.0614 | 0.5443 | 0.7848 |

Supplmentary Table 3. Characteristics of clusters of CHR sample

|  | Cluster 1 | Cluster 2 | Cluster 3 | chi, f | p |
| --- | --- | --- | --- | --- | --- |
|  | (N = 36) | (N = 109) | (N = 99) |  |  |
| Subgroups |  |  |  | 7.922 | 0.094 |
| BIPS | 3 | 3 | 6 |  |  |
| APS | 33 | 96 | 50 |  |  |
| GRDS | 0 | 10 | 7 |  |  |
| Presence of Religion (y/n) | 17/19 | 48/61 | 22/41 | 1.884 | 0.39 |
| Completion of military service (y/n) | 5/31 | 25/84 | 14/49 | 1.389 | 0.499 |
| Urbanicity |  |  |  | 6.956 | 0.138 |
| City | 25 | 78 | 55 |  |  |
| Small town | 8 | 24 | 5 |  |  |
| Rural area | 3 | 7 | 3 |  |  |
| Unemployment (y/n) | 8/28 | 30/79 | 5/58 | 9.403 | 0.009 |
| Male/Female | 30/6 | 65/44 | 47/16 | 8.691 | 0.013 |
| Family history of psychosis (y/n) | 3/33 | 19/90 | 13/50 | 2.537 | 0.281 |
| High parental socioeconomic status^a^ | 13/23 | 44/65 | 24/39 | 0.233 | 0.89 |
| Handedness (Right/Left) | 33/3 | 97/12 | 57/6 | 0.246 | 0.884 |
| Age | 19.8 (2.6) | 21.3 (4.6) | 20.3 (3.4) | 2.67 | 0.07 |
| Education Years | 12.5 (1.7) | 12.7 (2.1) | 12.7 (1.9) | 0.2 | 0.815 |
| IQ | 95.9 (10.5) | 102.9 (12.7) | 116.0 (11.2) | 38.75 | p < 0.001 |
| WCST perseverative error | 11.0 (4.3) | 9.5 (5.2) | 9.9 (7.3) | 0.91 | 0.406 |
| TMT-B (sec) | 82.4 (27.7) | 70.1 (25.6) | 58.5 (18.2) | 11.72 | p < 0.001 |
| Digit span | 11.3 (2.1) | 11.7 (2.6) | 12.9 (2.3) | 7.96 | 0.001 |
| TMT-A (sec) | 30.6 (16.2) | 26.0 (8.4) | 24.4 (7.9) | 4.48 | 0.013 |
| CVLT | 15.9 (5.5) | 22.7 (4.0) | 28.1 (2.9) | 105.45 | p < 0.001 |
| RCFT | 20.5 (5.8) | 23.7 (5.4) | 26.9 (5.3) | 16.32 | p < 0.001 |
| COWAT | 26.3 (12.8) | 32.2 (13.8 | 39 (13.3) | 10.78 | p < 0.001 |
| Category fluency task | 26.2 (12.2) | 29.5 (11.4) | 35.7 (11.4) | 9.41 | p < 0.001 |
| Strange Stories task | 19.4 (3.2) | 20.8 (3.1) | 22.5 (2.5) | 13.5 | p < 0.001 |
| HAM-A | 12.2 (6.8) | 10.3 (6.7) | 10.9 (7.5) | 1.01 | 0.366 |
| HAM-D | 14.3 (6.3) | 11.7 (6.9) | 12.9 (7.8) | 1.8 | 0.169 |
| GAFd | 48.9 (7.7) | 54.3 (9.2) | 55.2 (9.2) | 6.46 | 0.002 |
| % drop in GAF within 1 year | 30.3 (12.8) | 18.8 (11.9) | 11.2 (10.8) | 30.35 | p < 0.001 |
| SOPS positive symptom score | 11.3 (3.9) | 10.4 (3.9) | 9.1 (3.7) | 4.38 | 0.014 |
| SOPS negative symptom score | 14.6 (5.3) | 12.3 (6.2) | 12.9 (6.2) | 1.87 | 0.157 |
| SOPS disorganized symptom score | 5.4 (3.3) | 4.0 (2.7) | 3.9 (2.5) | 4.07 | 0.018 |
| SOPS general symptom score | 8.9 (4.5) | 6.8 (3.8) | 7.8 (3.9) | 4.63 | 0.011 |
| Social Functional Scale | 98.5 (9.7) | 99.9 (9.1) | 100.5 (8.4) | 0.6 | 0.5 |

BIPS, Brief Intermittent Psychotic Syndrome; APS, Attenuated Psychosis Symptoms Syndrome, GRD, Genetic Risk and Deterioration Syndrome; SOPS, Scale of Prodromal Symptoms; HAM-D, Hamilton Depression Rating Scale; HAM-A, Hamilton Anxiety Rating Scale; GAF, Global Assessment of Functioning; SFS, Social Functioning Scale, CVLT, California Verbal Learning Test; ROCF, Rey-Osterrieth Complex Figure Test; TMT, Trail Making Test; COWAT, Controlled Oral Word Association Test; WCST, Wisconsin Card Sorting Test; ^a,^ Scores of 1–3 indicate high status and scores of 4 –5 indicate low status.
